# Clinical outcomes prediction in kidney transplantation by use of biomarkers from hypothermic machine perfusion

**DOI:** 10.1590/S1677-5538.IBJU.2024.0166

**Published:** 2024-05-20

**Authors:** Ricardo Ribas de Almeida Leite, Maurilo Leite, Marcelo Einicker-Lamas, Rafael Hospodar Felippe Valverde, Luiz Carlos Duarte Miranda, Alberto Schanaider

**Affiliations:** 1 Departamento de Cirurgia Faculdade de Medicina Universidade Federal do Rio de Janeiro Rio de Janeiro RJ Brasil Programa de Pós-Graduação em Ciências Cirúrgicas, Departamento de Cirurgia, Faculdade de Medicina, Universidade Federal do Rio de Janeiro - UFRJ, Rio de Janeiro, RJ, Brasil;; 2 Programa de Pós-Graduação em Clínica Médica Faculdade de Medicina Universidade Federal do Rio de Janeiro Rio de Janeiro RJ Brasil Programa de Pós-Graduação em Clínica Médica, Disciplina de Nefrologia, Faculdade de Medicina, Universidade Federal do Rio de Janeiro - UFRJ, Rio de Janeiro, RJ, Brasil;; 3 Programa de Pós-Graduação em Fisiologia IBCCF Rio de Janeiro RJ Brasil Programa de Pós-Graduação em Fisiologia, Disciplina de Fisiologia, Instituto de Biofísica Carlos Chagas Filho - IBCCF, Rio de Janeiro, RJ, Brasil;; 4 Programa de Pós-Graduação em Biofísica IBCCF Rio de Janeiro RJ Brasil Programa de Pós-Graduação em Biofísica, Disciplina de Biofísica, Instituto de Biofísica Carlos Chagas Filho - IBCCF, Rio de Janeiro, RJ, Brasil;

**Keywords:** Kidney Transplantation, Delayed Graft Function, Graft Survival

## Abstract

**Purpose:**

The clinical outcomes of kidney transplantation from deceased donors have seen significant improvements with the use of machine perfusion (MP), now a standard practice in transplant centers. However, the use of perfusate biomarkers for assessing organ quality remains a subject of debate. Despite this, some centers incorporate them into their decision-making process for donor kidney acceptance. Recent studies have indicated that lactate dehydrogenase (LDH), glutathione S-transferase, interleukin-18, and neutrophil gelatinase-associated lipocalin (NGAL) could predict post-transplant outcomes.

**Materials and Methods:**

Between August 2016 and June 2017, 31 deceased-donor after brain death were included and stroke was the main cause of death. Pediatric patients, hypersensitized recipients were excluded. 43 kidneys were subjected to machine perfusion. Perfusate samples were collected just before the transplantation and stored at -80ºC. Kidney transplant recipients have an average age of 52 years, 34,9% female, with a BMI 24,6±3,7. We employed receiver operating characteristic analysis to investigate associations between these perfusate biomarkers and two key clinical outcomes: delayed graft function and primary non-function.

**Results:**

The incidence of delayed graft function was 23.3% and primary non-function was 14%. A strong association was found between NGAL concentration and DGF (AUC=0.766, 95% CI, P=0.012), and between LDH concentration and PNF (AUC=0.84, 95% CI, P=0.027). Other perfusate biomarkers did not show significant correlations with these clinical outcomes.

**Conclusion:**

The concentrations of NGAL and LDH during machine perfusion could assist transplant physicians in improving the allocation of donated organs and making challenging decisions regarding organ discarding. Further, larger-scale studies are required.

## INTRODUCTION

The shortage of donor organs, expanding waiting lists, and significant discard rates are central challenges in renal transplantation. The importance of organ quality in determining long-term function is increasingly recognized (1). The need to accept borderline grafts has led to the development and use of dynamic perfusion methods, aiming to assess, improve, and predict post-transplant outcomes (2). These higher-risk kidneys are more prone to preservation-induced injury, delayed graft function (DGF), primary non-function (PNF), and may have reduced long-term survival (3). Of note, a Brazilian multicenter study reported DGF incidences ranging from 29.9% to 87.7% across kidney transplant centers (4).

Hypothermic machine perfusion (HMP) is a technique developed in 1967 by Belzer, wherein the kidney is connected to a perfusion circuit, and a cooled preservation solution flows through the organ using a pump (5, 6). This method benefits from a significant reduction in cellular metabolism, down to 5-10%, due to the low temperatures. Additionally, it maintains hemodynamic stimulus, improving renal cortical microcirculation during preservation (5). In the United States, 32 to 38% of kidneys from brain-dead donors for transplantation are stored using machine perfusion (7). A Brazilian multicenter trial, comparing machine perfusion to cold storage, showed a lower incidence of DGF in the HMP group (45 vs 61%) (8). Our group conducted a systematic review and meta-analysis, which aligns with international findings, concluding that HMP reduces DGF in brain-dead donors (9).

Over the years, transplant community has sought ways to improve performance and increase graft survival. In this sense, the search for non-invasive markers that can predict unfavorable clinical outcomes has become a relevant area of research. Accurate evaluation of kidney quality may reduce the discard of viable kidneys and the transplant of poor-quality kidneys with unacceptable survival rates (10).

We studied four perfusate biomarkers, linked to renal cellular injury. Lactate dehydrogenase (LDH) as a general marker of cellular injury. LDH release to assess overall renal injury, given that perfusate samples are from an isolated kidney on the pump (11). Glutathione S-transferase (GST), an enzyme found in renal tubules which plays a role in waste product deconjugation and excretion into urine (12). Interleukin-18 (IL-18), an inflammation marker, predictive of DGF when measured in urine (13). Neutrophil gelatinase-associated lipocalin (NGAL), part of the lipocalin family and initially identified in neutrophils, due to its association with DGF and PNF in kidney injury (14). In this sense, our group investigated associations of these perfusate biomarkers with delayed graft function and primary non-function in kidney transplants.

## MATERIALS AND METHODS

This is a multicenter, observational, retrospective, and translational study. The study adheres to the ethical standards and guidelines of the Brazilian transplant centers and received approval from the Ethics and Research Committee (CEP) under the IRB number: 4.355.970.

From August 2016 to June 2017, 31 deceased donors of brain death (DBD) who underwent hypothermic machine perfusion (HMP) were included in the data analysis by the organ procurement organization (OPO). All patients came from hospitals located in the State of Rio de Janeiro, Brazil. Pediatric donors, stored in cold ischemia (CS), and kidneys without collected perfusate samples were excluded. Recipients who came to death within three months post-transplantation were also excluded, as accurate clinical outcome analysis was not feasible. Patients who were hypersensitive or underwent retransplantation were excluded due to potential variations in immunosuppression affecting data interpretation. There were 43 kidneys transplant recipients.

The LifePort Kidney Transporter (Organ Recovery Systems, Itasca, IL) was used for all individually perfused kidneys. The kidneys were pumped using pulsatile flow with 1 liter of kidney perfusion solution (KPS-1) at a targeted pressure of 30 mmHg and temperature of 4ºC (15). Upon arrival and before inspection by the transplant surgeon, 20 mL of perfusate was collected from the renal vein and stored at -20ºC. The samples were transported on ice and stored at -80ºC without protease inhibitors until biomarker measurement.

Samples stored at -80ºC were thawed, brought to room temperature, and diluted 50-fold with dilution buffers for perfusate biomarker analysis. IL-18 (RAB0543 – Sigma-Aldrich, San Luis, MI), NGAL (RAB0332 Sigma-Aldrich San Luis, MI), LDH (MK066 Sigma-Aldrich, San Luis, MI), and GST (CS0410 Sigma-Aldrich, San Luis, MI) levels were measured using enzyme-linked immunosorbent assay (ELISA) kits. The ELISA plates were read using a SpectraMax M5 plate reader (Molecular Devices, San Jose CA).

The clinical outcomes assessed were delayed graft function (DGF) and primary non-function (PNF). DGF was defined as the necessity for dialysis within the first seven days post-transplant, and PNF as complete graft non-function three months post-transplant, in the absence of rejection or surgical factors, necessitating dialysis (10, 16).

### Statistical Analysis

Data were analyzed using SPSS 21 statistical package software. Descriptive statistics, including percentages, means (±SD), medians [IQR], and frequencies, were presented in tables and graphs. To associate perfusate biomarkers with clinical outcomes, Spearman correlations, the Wilcoxon test, and the Chi-square test were used. Receiver operating curve (ROC) and area under the curve (AUC) analyzes identified the best clinical predictors, allowing graphical analysis of sensitivity and false positives. A P-value of <0.05 was considered statistically significant.

## RESULTS

A total of 31 deceased brain death donors (DBD) were included. From these, 43 kidneys stored using machine perfusion, had perfusate samples collected for biomarkers measurement, and were subsequently transplanted. The clinical and demographic characteristics of the donors are summarized in Table-1. The mean age of the donors was 54.8±14.1 years, with a majority of right-sided 26 out of 31 grafts (60.5%). The kidney donor profile index (KDPI) was 73.1±24.7. Stroke was the predominant cause of death 35 of 43 donors (81.4%), and none had diabetes.

**Table 1:** Donor clinical and demographic characteristics.

Characteristic		Total n=43	DGF n=10 (23.3%)	PNF n=6 (14.0%)
Donors		31	9	5
Laterality	Right	26(60.5%)	6(60.0%)	4(66.7%)
	**Left**	17(39.5%)	4(40.0%)	2(33.3%)
**Age (years)**	**Mean±sd**	54.8±14.1	51.2±18.8	61.7±12.2
	**Median [IQR]**	56[51-65]	54[45-67]	66[54-70]
**Race**	**White**	23(53.5%)	6(60%)	2(33.3%)
	**Mixed race**	9(20.9%)	3(30%)	-----
	**Afrodescendant**	11(25.9%)	1(10%)	4(66.7%)
**BMI^a^**	**Mean±sd**	28.6±5.3	28.4±4.5	29.9±5.7
	**Median [IQR]**	27.3[25.1-29.4]	27.9[25.0-31.2]	28.9[24.5-35.5]
	**<25**	9(20.9%)	2(20.0%)	2(33.3%)
	**25-29**	25(58.2%)	5(50.0%)	1(16.7%)
	**≥30**	9(20.9%)	3(30.0%)	3(50.0%)
**KDPI (%)^b^**	**Mean±sd**	73.1±24.7	68.0±30.4	89.3±14.2
	**Median [IQR]**	83[61-93]	71[52-94]	93[82-100]
**KDRI^c^**	**Mean±sd**	1.40±0.44	1.34±0.47	1.78±0.48
	**Median [IQR]**	1.40[1.11-1.67]	1.23[1.03-1.74]	1.68[1.43-2.34]
**Hypertension**		11(25.6%)	3(30.0%)	3(50.0%)
**Diabetes**		-----	-----	-----
**HCV^d^**		4(9.3%)	-----	1(16.7%)
**Creatinine level at enrollment**	**Mean±sd**	1.10±0.39	0.99±0.35	1.42±0.38
	**Median [IQR]**	1.10[0.80-1.40]	1.05[0.68-1.18]	1.50[1.10-1.68]
**Terminal serum creatinine**	**Mean±sd**	2.08±1.58	2.83±2.15	1.83±0.56
	**Median [IQR]**	1.70[1.20-2.30]	1.95[1.50-3.50]	2.0[1.30-2.30]
**Causa mortis**	**Cerebral Trauma**	6(14.0%)	2(20.0%)	-----
	**Stroke**	35(81.4%)	8(80.0%)	5(83.3%)
	**Intracranial Hypertension**	1(2.3%)	-----	-----
	**Anoxic Encephalopathy**	1(2.3%)	-----	1(16.7%)

Values are mean ± standard derivation. median [interquartile range] or n (%). All kidneys are derived from deceased donor after brain death; DGF. delayed graft function; PNF. primary nonfunction. a BMI body mass index; b KDPI kidney donor profile index; c KDRI kidney donor risk index; d HCV hepatitis c virus

The characteristics of the 43 kidney transplant recipients are detailed in Table-2. Hypertensive kidney disease was the most common cause of ESRD 17 out of 43 recipients (39.5%). The surgical transplants were done in two transplant centers following the same clinical protocols. All patients received identical induction and maintenance immunosuppression, mitigating potential bias. The mean recipient age was 52.8±13.1 years. The ethnic distribution was balanced, with 14 (32.6%) white, 14 (32%) mixed race, and 15 (34.8%) recipients afro-descendants, and the majority were male 28 of 43 (65.1%). The median cold ischemia time was 19.6 hours [16.1-23.6]. Most patients had undergone hemodialysis 41 out of 43 (95.3%). Additionally, includes information on the antibody reactivity panel (PRA), the human leukocyte antigen system (HLA), and an average mismatch of 3.2±1.1.

**Table 2:** Recipient demographic and clinical characteristics.

Characteristics		Total n=43	DGF n=10 (23.3%)	PNF n=6 (14.0%)
Transplant Center	1	37(86.0%)	8(80.0%)	6(100%)
	**2**	6(14.0%)	2(20.0%)	-----
**Year of kidney transplantation**	**2016**	24(55.8%)	8(80.0%)	3(50.0%)
	**2017**	19(44.2%)	2(20.0%)	3(50.0%)
**Age (years)**	**Mean±sd**	52.8±13.1	51.0±13.8	52.5±10.4
	**Median [IQR]**	52[42-60]	50[42-62]	53[46-62]
**Ethnicity**	**White**	14(32.6%)	4(40.0%)	3(50.0%)
	**Mixed**	14(32.6%)	4(40.0%)	2(33.3%)
	**Afrodescendant**	15(34.8%)	2(20.0%)	1(16.7%)
**Gender**	**Male**	28(65.1%)	8(80.0%)	5(83.3%)
	**Female**	15(34.9%)	2(20.0%)	1(16.7%)
**Smoking**		9(20.9%)	5(50.0%)	1(16.7%)
**BMI^a^**	**Mean±sd**	24.6±3.7	25.1±4.3	25.7±3.4
	**Median [IQR]**	24.0[21.9-28.1]	23.7[21.4-29.1]	26.3[22.0-28.8]
	**<25**	25(58.1%)	5(50.0%)	3(50.0%)
	**25-29**	13(30.3%)	3(30.0%)	3(50.0%)
	**≥30**	5(11.6%)	2(20.0%)	-----
**Blood type**	**O**	17(39.5%)	4(40.0%)	2(33.3%)
	**A**	18(41.9%)	4(40.0%)	3(50.0%)
	**B**	7(16.3%)	2(20.0%)	1(16.7%)
	**AB**	1(2.3%)	-----	-----
**Cause of end-stage renal disease**	**Glomerulonephritis**	10(23.3%)	3(30.0%)	1(16.7%)
	**Hypertension**	17(39.5%)	3(30.0%)	3(50.0%)
	**HIV ^b^**	1(2.3%)	1(10.0%)	-----
	**Diabetes**	7(16.3%)	2(20.0%)	2(33.3%)
	**Polycistic Kidney Disease**	3(7.0%)	1(10.0%)	-----
	**Infection**	1(2.3%)	-----	-----
	**Lithiasis**	1(2.3%)	-----	-----
	**Berger’s Disease**	1(2.3%)	-----	-----
**HBV^c^**		1(2.3%)	1(10.0%)	-----
**HIV**		3(7.0%)	1(10.0%)	-----
**HCV^d^**		3(7.0%)	2(20.0%)	-----
**CMV^e^**		40(93.0%)	9(90.0%)	6(100%)
**Toxoplasmosis**		41(95.3%)	9(90.0%)	6(100%)
**PRAC I^f^ >20**		8(18.6%)	3(30.0%)	1(16.7%)
**PRAC II >20**		5(11.6%)	-----	1(16.7%)
**Dialysis**	**HD^g^**	41(95.3%)	9(90.0%)	6(100%)
	**PD^h^**	2(4.7%)	1(10.0%)	-----
**HLA_A1^i^**	**Mean±sd**	10.9±13.9	7.5±11.6	5.5±9.1
	**Median [IQR]**	3[2-23]	2[2-10]	2[1-8]
**HLA_A2**	**Mean±sd**	28.6±19.2	24.3±19.9	31.3±23.5
	**Median [IQR]**	30[23-33]	26[2-32]	30[18-41]
**HLA_B1**	**mean±sd**	21.4±14.9	20.5±13.5	22.7±16.0
	**Median [IQR]**	14[8-35]	15[8-36]	15[12-43]
**HLA_B2**	**mean±sd**	43.5±13.1	40.6±11.5	42.8±14.1
	**Median [IQR]**	45[39.5-52]	44[37-46]	47[37-52]
**HLA_DR1**	**mean±sd**	6.7±4.3	7.8±5.1	6.2±4.9
	**Median [IQR]**	7[3-11]	7[3-13]	6[3-9]
**HLA_DR2**	**mean±sd**	13.3±5.7	16.4±10.3	11.8±2.4
	**Median [IQR]**	13[11-15]	10[11-15]	12[10-14]
**MISMATCH**	**Mean±sd**	3.2±1.1	3.1±1.1	2.8±1.2
	**Median [IQR]**	3[2-4]	3[3-4]	3[2-4]
**Transfusions**	**mean±sd**	1.6±1.1	1.2±1.4	0.3±0.8
	**Median [IQR]**	0[0-2]	0[0-2]	0[0-1]
**Cold ischemic time (hr)**	**mean±sd**	19.5±4.8	21.0±4.2	20.6±5.5
	**Median [IQR]**	19.6[16.1-23.6]	20.3[17.1-25.0]	23.0[14.7-24.5]

Values are mean ± standard derivation. median [interquartile range] or n (%). n=43 recipients; ^a^ BMI body mass index; ^b^ HIV human immunodeficiency virus; ^c^ HBV hepatitis B virus; ^d^ HCV hepatitis c virus; ^e^ CMV cytomegalovirus; ^f^ PRA panel-reactive antibody; ^g^ HD hemodialysis; ^h^ PD peritoneal dialysis; ^I^ HLA human leukocyte anti.

The relationship between four perfusate biomarkers (IL-18, GST, LDH, NGAL) and clinical outcomes (DGF and PNF) was evaluated using receiver-operator curves (ROC) and calculating the area under the curve (AUC). An AUC above 0.70 indicates satisfactory performance, while below 0.50 suggests inadequacy in discriminating the association of parameters. IL-18, with an AUC of 0.295, was excluded from further analysis (Figures 1A and 1B).

**Figures:**
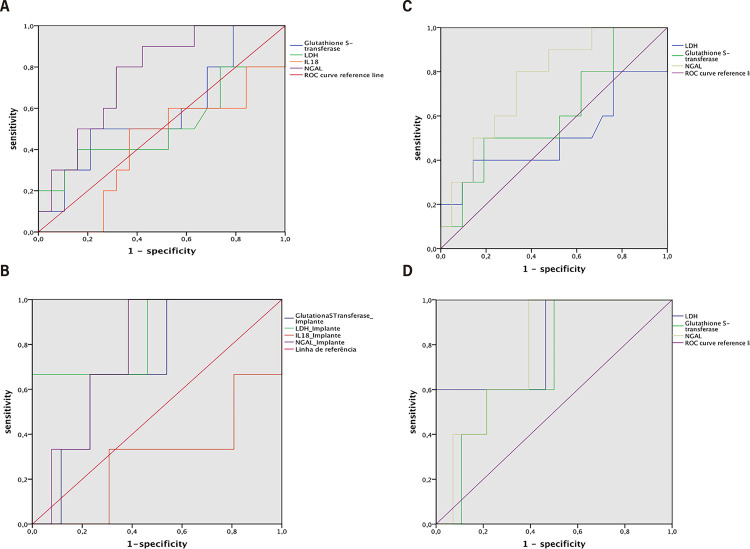
: 1A) ROC of four biomarkers (GST, IL-18, LDH and NGAL) and their association with DGF. AUC of IL-18 is under 0.5 (0.421), therefore no validity in the analysis of this biomarker and the clinical outcome studied, being excluded from the statistics; B) ROC of four biomarkers (GST, IL-18, LDH and NGAL) and their association with PNF. AUC of IL-18 is under 0.5 (0.295), therefore no validity in the analysis of this biomarker and the clinical outcome studied, being excluded from the statistics; C) ROC of three biomarkers (GST, LDH and NGAL) and their association with DGF. NGAL concentrations in perfusate samples showed greatest efficacy on predicting DGF among the biomarkers analyzed. (AUC= 0.766, IC 95% 0.603-0.929, P=0.012); D) ROC of three biomarkers (GST, LDH and NGAL) and their association with PNF. LDH concentrations in perfusate samples showed greatest efficacy on predicting PNFF among the biomarkers analyzed. (AUC=0.814, IC 95% 0.598-1.000, P=0.027).

The incidence of DGF affected 10 out of 43 patients (23.3%) requiring dialysis in the first week post-transplant. NGAL emerged as the most reliable predictive biomarker for DGF, with statistical significance (AUC=0.766, 95% CI 0.603-0.929, P=0.012). LDH and GST showed no significant correlation with DGF (Figure-1C).

For primary non-function (PNF), 6 out of 43 recipients (14%) experienced graft failure within three months post-transplant. The AUC for predicting PNF using LDH levels in perfusate was 0.84 (95% CI 0.598-1.000, P=0.027), suggesting a significant association. Other biomarkers did not show a significant association with PNF (Figure-1D).

## DISCUSSION

HMP not only protects isolated allografts but also provides an opportunity for observation and intervention. The real-time parameters and the ability to extract or add perfusate have become a new focus for transplant physicians (17, 18). Over ten biomarkers have been reported internationally in perfusate studies (16). Our group conducted the first Brazilian multicenter study correlating perfusate biomarkers (LDH, IL-18, NGAL, and GST) with clinical outcomes (DGF and PNF) in kidney transplant patients.

The incidence of DGF varies across continents. In the United States, the incidence is around 30%, including donations after circulatory death (DCD) (19, 20). In European centers, it ranges from 30 to 35% (21, 22), and between 35 to 45% in countries participating in the European Senior Transplant Program (23, 24). In Australia and New Zealand, the incidence is around 25% (25). A recent Brazilian multicenter study reported DGF incidences ranging from 29.9% to 87.7% among centers (4). In our study, a 23.3% incidence of DGF was observed, presumably lowered by the use of HMP in all donors.

In 2000, David Goetz, a graduate student at the University of California in San Francisco, under the mentorship of Professor Roland Strong, first described the three-dimensional structure of NGAL, showing high similarity to the lipocalins protein superfamily (26). NGAL is now regarded as the “troponin of the kidney” (27, 28). In the renal transplant setting, NGAL has been identified as a valuable tool for monitoring allograft function, particularly in the early postoperative period (29). Several studies have shown that NGAL detected in graft perfusate at the end of CIT correlates positively with donor age and last measured serum creatinine, both known risk factors for DGF (30, 31). Our study also sought to correlate four perfusate biomarkers with DGF using ROC analysis, finding NGAL as the biomarker that significantly correlated with DGF (AUC=0.766, 95% CI 0.603-0.929, P=0.012).

Primary non-function (PNF), defined as dialysis dependence for 3 months post-transplant, is a rare but severe outcome (32). GST and LDH were the most common biomarkers associated with PNF (33). Studies have shown LDH’s distribution in normal renal tissue and its elevation in serum and urine in patients with renal diseases (34, 35). Our study evaluated the correlation of perfusate biomarkers with PNF, finding LDH to be the only biomarker significantly associated with PNF (AUC=0.84, 95% CI 0.598-1000, P=0.027). These findings need confirmation from larger studies that include clinical and hemodynamic data.

Predicting clinical outcomes non-invasively and avoiding transplants with poor prognosis is a key goal in transplantation research. The ideal biomarker for renal transplant settings, which is non-invasive, safe, low-cost, and highly sensitive and specific, has not yet been identified (29). Our study analyzed four biomarkers from HMP and their connection with post-transplant outcomes. These findings are relevant as they point to specific biomarkers that can be used in our transplant procedures using HMP, in order to predict or prepare for high possibilities of DGF and PNF in transplanted patients.

The use of hypothermic machine perfusion is still restricted to few Brazilian centers, and therefore an insufficient number of national publications. This study is the first to evaluate the correlation of four perfusate biomarkers with clinical outcomes in our population. Due to the high rates of DGF exposed by recent articles (4), it is imperative to develop ways to improve prognosis in transplant patients.

Nevertheless, this study has some limitations including a small sample size, low rates of DGF and PNF, and lack of evaluation of pump parameters like vascular resistance and cost effectiveness.

## CONCLUSIONS

Our study suggests that NGAL could be a potential biomarker for predicting DGF, and LDH might play a similar role in relation to PNF. The ongoing research to access and validate donor kidney quality is crucial for transplant physicians to improve long-term graft outcomes and reduce retransplantation.

## References

[1] Ponticelli C, Reggiani F, Moroni G (2022). Delayed Graft Function in Kidney Transplant: Risk Factors, Consequences and Prevention Strategies. J Pers Med.

[2] Ghoneima AS, Sousa Da Silva RX, Gosteli MA, Barlow AD, Kron P (2023). Outcomes of Kidney Perfusion Techniques in Transplantation from Deceased Donors: A Systematic Review and Meta-Analysis. J Clin Med.

[3] Jochmans I, O'Callaghan JM, Pirenne J, Ploeg RJ (2015). Hypothermic machine perfusion of kidneys retrieved from standard and high-risk donors. Transpl Int.

[4] de Sandes-Freitas TV, Mazzali M, Manfro RC, de Andrade LGM, Vicari AR, de Sousa MV (2021). Exploring the causes of the high incidence of delayed graft function after kidney transplantation in Brazil: a multicenter study. Transpl Int.

[5] Tatsis V, Dounousi E, Mitsis M (2021). Hypothermic Machine Perfusion of Kidney Transplant: A Mini-Review. Transplant Proc.

[6] Karangwa SA, Dutkowski P, Fontes P, Friend PJ, Guarrera JV, Markmann JF (2016). Machine Perfusion of Donor Livers for Transplantation: A Proposal for Standardized Nomenclature and Reporting Guidelines. Am J Transplant.

[7] Malinoski D, Saunders C, Swain S, Groat T, Wood PR, Reese J (2023). Hypothermia or Machine Perfusion in Kidney Donors. N Engl J Med.

[8] Tedesco-Silva Junior, Mello Offerni JC, Ayres Carneiro V, Ivani de Paula M, Neto ED, Brambate Carvalhinho Lemos F (2017). Randomized Trial of Machine Perfusion Versus Cold Storage in Recipients of Deceased Donor Kidney Transplants With High Incidence of Delayed Graft Function. Transplant Direct.

[9] Leite RRA, Schanaider A, da-Fonseca ER, Xavier VL, de-Miranda LCD (2019). Machine perfusion versus cold storage in renal preservation of deceased donors with brain death: systematic review and meta-analysis. Rev Col Bras Cir.

[10] Parikh CR, Hall IE, Bhangoo RS, Ficek J, Abt PL, Thiessen-Philbrook H (2016). Associations of Perfusate Biomarkers and Pump Parameters With Delayed Graft Function and Deceased Donor Kidney Allograft Function. Am J Transplant.

[11] Moers C, Varnav OC, van Heurn E, Jochmans I, Kirste GR, Rahmel A (2010). The value of machine perfusion perfusate biomarkers for predicting kidney transplant outcome. Transplantation.

[12] Beckett GJ, Hayes JD (1993). Adv Clin Chem.

[13] Parikh CR, Jani A, Mishra J, Ma Q, Kelly C, Barasch J (2006). Urine NGAL and IL-18 are predictive biomarkers for delayed graft function following kidney transplantation. Am J Transplant.

[14] Kjeldsen L, Johnsen AH, Sengeløv H, Borregaard N (1993). Isolation and primary structure of NGAL, a novel protein associated with human neutrophil gelatinase. J Biol Chem.

[15] Hall IE, Bhangoo RS, Reese PP, Doshi MD, Weng FL, Hong K (2014). Glutathione S-transferase iso-enzymes in perfusate from pumped kidneys are associated with delayed graft function. Am J Transplant.

[16] Qiao Y, Ding C, Li Y, Tian X, Tian P, Ding X (2021). Predictive value of hypothermic machine perfusion parameters combined perfusate biomarkers in deceased donor kidney transplantation. Chin Med J (Engl).

[17] De Deken J, Kocabayoglu P, Moers C (2016). Hypothermic machine perfusion in kidney transplantation. Curr Opin Organ Transplant.

[18] Ding CG, Tian PX, Ding XM, Xiang HL, Li Y, Tian XH (2018). Beneficial Effect of Moderately Increasing Hypothermic Machine Perfusion Pressure on Donor after Cardiac Death Renal Transplantation. Chin Med J (Engl).

[19] Irish WD, Ilsley JN, Schnitzler MA, Feng S, Brennan DC (2010). A risk prediction model for delayed graft function in the current era of deceased donor renal transplantation. Am J Transplant.

[20] Taber DJ, DuBay D, McGillicuddy JW, Nadig S, Bratton CF, Chavin KD (2017). Impact of the New Kidney Allocation System on Perioperative Outcomes and Costs in Kidney Transplantation. J Am Coll Surg.

[21] Gavela Martínez E, Pallardó Mateu LM, Sancho Calabuig A, Beltrán Catalán S, Kanter Berga J, Ávila Bernabeu AI (2011). Delayed graft function after renal transplantation: an unresolved problem. Transplant Proc.

[22] Fonseca I, Teixeira L, Malheiro J, Martins LS, Dias L, Castro Henriques A (2015). The effect of delayed graft function on graft and patient survival in kidney transplantation: an approach using competing events analysis. Transpl Int.

[23] Frei U, Noeldeke J, Machold-Fabrizii V, Arbogast H, Margreiter R, Fricke L (2008). Prospective age-matching in elderly kidney transplant recipients--a 5-year analysis of the Eurotransplant Senior Program. Am J Transplant.

[24] Schachtner T, Otto NM, Reinke P (2019). Two decades of the Eurotransplant Senior Program: the gender gap in mortality impacts patient survival after kidney transplantation. Clin Kidney J.

[25] Vacher-Coponat H, McDonald S, Clayton P, Loundou A, Allen RD, Chadban SJ (2013). Inferior early posttransplant outcomes for recipients of right versus left deceased donor kidneys: an ANZDATA registry analysis. Am J Transplant.

[26] Goetz DH, Willie ST, Armen RS, Bratt T, Borregaard N, Strong RK (2000). Ligand preference inferred from the structure of neutrophil gelatinase associated lipocalin. Biochemistry.

[27] Ronco C, Legrand M, Goldstein SL, Hur M, Tran N, Howell EC (2014). Neutrophil gelatinase-associated lipocalin: ready for routine clinical use? An international perspective. Blood Purif.

[28] Devarajan P (2008). Neutrophil gelatinase-associated lipocalin (NGAL): a new marker of kidney disease. Scand J Clin Lab Invest Suppl.

[29] Cappuccilli M, Capelli I, Comai G, Cianciolo G, La Manna G (2018). Neutrophil Gelatinase-Associated Lipocalin as a Biomarker of Allograft Function After Renal Transplantation: Evaluation of the Current Status and Future Insights. Artif Organs.

[30] van den Akker EK, Hesselink DA, Manintveld OC, IJzermans JN, de Bruijn RW, Dor FJ (2015). Neutrophil Gelatinase-Associated Lipocalin, but Not Kidney Injury Marker 1, Correlates with Duration of Delayed Graft Function. Eur Surg Res.

[31] Qurashi S, Ghamdi G, Jaradat M, Tamim H, Aljumah A, Tamimi W (2014). Urinary neutrophil gelatinase-associated lipocalin and the occurrence of delayed graft function after kidney transplant. Exp Clin Transplant.

[32] Mohan S, Reese P, Hall I, Doshi M, Jia Y, Philbrook HT, al t. (2019). Primary Nonfunction after Kidney Transplantation is Associated with Elevated Donor Urinary Biomarkers. Am J Transplant.

[33] Guzzi F, Knight SR, Ploeg RJ, Hunter JP (2020). A systematic review to identify whether perfusate biomarkers produced during hypothermic machine perfusion can predict graft outcomes in kidney transplantation. Transpl Int.

[34] Kang SK, Ha CY, Cho KH, Park SK, Kim UH (1991). Changes of lactate dehydrogenase and its isoenzyme activity in renal diseases. Nephron.

[35] Zager RA, Johnson AC, Becker K (2013). Renal Cortical Lactate Dehydrogenase: A Useful, Accurate, Quantitative Marker of In Vivo Tubular Injury and Acute Renal Failure. PLoS One.

